# Prognostic value of protein tyrosine kinase 6 (PTK6) for long-term survival of breast cancer patients

**DOI:** 10.1038/sj.bjc.6604660

**Published:** 2008-09-09

**Authors:** M Aubele, A K Walch, N Ludyga, H Braselmann, M J Atkinson, B Luber, G Auer, S Tapio, T Cooke, J M S Bartlett

**Affiliations:** 1 Institute of Pathology, Helmholtz Centre Munich, German Research Centre for Environmental Health, Neuherberg D-85764, Germany; 2 Institute of Molecular Radiation Biology, Helmholtz Centre Munich, German Research Centre for Environmental Health, Neuherberg D-85764, Germany; 3 Institute of Radiation Biology, Helmholtz Centre Munich, German Research Centre for Environmental Health, Neuherberg D-85764, Germany; 4 Klinik für Strahlentherapie, Technische Universität München, Ismaninger Strasse 22, München 81675, Germany; 5 Institut für Pathologie, Technische Universität München, Trogerstr. 18, München 81675, Germany; 6 Department of Oncology and Pathology, Karolinska Institute and Hospital, Stockholm S-17176, Sweden; 7 University Department of Surgery, Glasgow Royal Infirmary, Queen Elizabeth Building, 10 Alexandra Parade, Glasgow G31 2ER, UK; 8 Endocrine Cancer Group, Cancer Research Centre, Western General Hospital, Crewe Road South, Edinburgh EH4 2XR, UK

**Keywords:** PTK6 (BRK), breast cancer, prognosis, MAPK, Sam68, PTEN

## Abstract

The cytoplasmic tyrosine kinase PTK6 (BRK) shows elevated expression in approximately two-thirds of primary breast tumours, and is implicated in EGF receptor-dependent signalling and epithelial tumorigenesis. Using immunohistochemistry, we performed a retrospective study on 426 archival breast cancer samples from patients with long-term follow-up and compared the protein expression levels of PTK6, the HER receptors, Sam68 (a substrate of PTK6), and signalling proteins including MAP kinase (MAPK), phosphorylated MAPK (P-MAPK), and PTEN. We show that PTK6 expression is of significant prognostic value in the outcome of breast carcinomas. In multivariate analysis, the disease-free survival of patients of ⩾240 months was directly associated with the protein expression level of PTK6 (*P*⩽0.001), but was also inversely associated with nodal status (*P*⩽0.001) and tumour size (*P*⩽0.01). PTK6 expression in tumour tissue significantly correlated (*P*⩽0.05) with the expression of PTEN, MAPK, P-MAPK, and Sam68. To investigate whether these correlations may be due to molecular interactions between PTK6 and these proteins, we used protein extracts from the T47D cell line for immunoprecipitation and western blot analysis. By this, interactions could be demonstrated between PTK6 and MAPK, P-MAPK, HER2/neu, HER3, HER4, PTEN, and Sam68. On the basis of these results, we suggest that PTK6 may serve as a future target for the development of novel treatments in breast cancer.

The cytoplasmic protein tyrosine kinase PTK6 (BRK, breast tumour kinase) was originally cloned from a human metastatic breast tumour (Mitchell *et al*, 1994). PTK6 was shown to be overexpressed in two-thirds of breast carcinomas (Mitchell *et al*, 1994; Barker *et al*, 1997; Llor *et al*, 1999; Born *et al*, 2005; Aubele *et al*, 2007; Ostrander *et al*, 2007), and expression is elevated in colon tumours and several cancer cell lines (Barker *et al*, 1997; Llor *et al*, 1999; Kamalati *et al*, 2000; Meric *et al*, 2002; Derry *et al*, 2003).

PTK6 possesses sequences with predicted homology with Src domains (SH3 and SH2) (Kamalati *et al*, 1996; Zhang *et al*, 2005), suggesting interactions with other signalling proteins. On account of the known involvement of Src in epithelial tumour development and structural similarities with Src, PTK6 is thought to have a function in epithelial tumorigenesis (Petro *et al*, 2004; Zhang *et al*, 2005).

Several *in vitro* studies have attempted to identify the physiological function and the interaction partners of PTK6. Many of these suggest a possible involvement of PTK6 in modulating signal transduction of HER receptor tyrosine kinases (Kamalati *et al*, 1996, 2000; Chen *et al*, 2004; Haegebarth *et al*, 2005; Zhang *et al*, 2005). Knockdown of PTK6 decreases proliferation in breast cancer cell lines (Harvey and Crompton, 2003) and blocks activity of a GTPase, of ERK5 (extracellular signal-regulated kinase) and p38 mitogen-activated protein kinase (MAPK), but not Akt (Mitchell *et al*, 1994; Barker *et al*, 1997; Llor *et al*, 1999; Born *et al*, 2005; Ostrander *et al*, 2007). Furthermore, PTK6 phosphorylates STAT3 (signal transducer and activator of transcription 3; Liu *et al*, 2006) and STAT 5b, leading to increased STAT 5b transcriptional activity in several breast cancer cell lines (Weaver and Silva, 2007).

In recent years, several additional PTK6 substrates have been identified (Derry *et al*, 2000; Babic *et al*, 2004; Haegebarth *et al*, 2004, 2005). The first reported substrate of PTK6 phosphorylation was Sam68 (Src-associated in mitosis 68 kDa), and it was shown that PTK6 negatively regulates its RNA-binding activity (Derry *et al*, 2000). This may have an impact on the post-transcriptional regulation of gene expression.

Besides these important *in vitro* findings, PTK6's function *in vivo* remains largely undefined. Very little is known about its physiological relevance in inducing phosphorylation events, in particular in tumour tissue. As mentioned above, there is increasing evidence that PTK6 is involved in EGF receptor signalling (Kamalati *et al*, 1996; Born *et al*, 2005; Zhang *et al*, 2005). If true, this is a most important finding, as the HER receptors–in particular HER2/neu–are of therapeutic and prognostic significance in breast cancer (Slamon, 1990; Meric *et al*, 2002; Hudelist *et al*, 2003; Witton *et al*, 2003; Abd El-Rehim *et al*, 2004; Bianchi *et al*, 2006). The HER2/neu antibody trastuzumab induces tumour regression in approximately 30 – 35% of patients with HER2-amplified metastatic breast cancer (Hsieh and Moasser, 2007), indicating that additional signalling molecules may influence the biological response to trastuzumab. Cytoplasmic tyrosine kinases, such as PTK6, containing SH2 and SH3 domains, are thought to modify receptor tyrosine kinase signalling (Petro *et al*, 2004; Zhang *et al*, 2005).

The data concerning PTK6 expression in tumour tissues are, so far, very limited. PTK6 was found to be localised in the nucleus and cytoplasm in normal oral epithelium, and it was found in perinuclear regions in poorly differentiated oral squamous carcinomas (Petro *et al*, 2004). In normal breast epithelium, PTK6 expression is low or undetectable, but it is elevated in many breast carcinomas (Petro *et al*, 2004; Zhang *et al*, 2005; Aubele *et al*, 2007), indicating that PTK6 overexpression may be related to carcinogenesis. In contrast, high levels of PTK6 are expressed in some differentiating epithelial tissues, such as normal gastrointestinal tract, skin (Llor *et al*, 1999; Haegebarth *et al*, 2004), and prostate (Derry *et al*, 2003). Moreover, PTK6 expression is associated with the degree of differentiation of breast tissue as indicated by oestrogen receptor (ER) expression (Zhao *et al*, 2003).

As recently reported by us, PTK6 protein expression has prognostic value in a small set of 105 breast carcinomas (Aubele *et al*, 2007), and correlates with the expression of HER receptors (Born *et al*, 2005; Aubele *et al*, 2007). We have extended the study to a total of 426 patients with a long-term follow-up. As PTK6 seems to be implicated in signalling pathways, we have expanded our immunohistochemical analysis of PTK6 to its nuclear substrate Sam68, the signalling molecules MAPK and P-MAPK, and the tumour suppressor protein PTEN.

## Materials and methods

### Patients and tumour samples

Immunohistochemical studies were performed on formalin-fixed, paraffin-embedded tissues from invasive breast carcinomas. The study comprises 426 patients with a detailed long-term follow-up, including those cases reported previously (Aubele *et al*, 2007). Two hundred forty-four of the tumours were lymph node negative, 182 were node positive, and the majority of the tumours in this study were <2 cm in size (*n*=234). The hormone receptor status was evaluated immunohistochemically. Classification into negative (no or low) and positive (medium or high staining intensity) revealed that 311 and 141 tumours were ER positive and progesterone receptor (PrR) positive, respectively. Histological classification (WHO 2003) defined 332 tumours as being invasive ductal not otherwise specified (NOS). The remaining cases were classified as tubular (14), lobular (47), medullary (2), and others (31). A total of 221 cases were classified as grade 2, 109 cases as grade 3, and 96 cases as grade 1, according to Elston and Ellis (1991).

All patients were surgically treated, and none of the patients received preoperative treatment. Fifty patients received no postoperative treatment, whereas adjuvant antihormonal treatment was administered to 203 patients, radio- and/or chemotherapy to 33 patients, and 140 patients received combined treatment regimes (radio- and/or chemo- and/or antihormonal treatment).

The median clinical follow-up of patients was 80 months (mean 94, maximum 264 months) with 121 (28%) of the patients relapsing with distant metastases and/or local recurrence within the total follow-up period. Ethical approval for the study was obtained from the Ethics Committee of the medical faculty of the Technical University of Munich.

### Tissue microarrays (TMA)

Tissue microarray construction was performed using the same quality criteria and methodology as described with a tissue-arraying instrument (Beecher Instruments Inc., Silver Spring, MD, USA) (Aubele *et al*, 2007). 5 *μ*m sections were cut from the TMA blocks, and both the TMA and punched block were re-examined to validate representative sampling.

### Immunohistochemistry (IHC)

For IHC on the TMA sections, the antibodies for HER1, HER2/neu, HER3, HER4, and PTK6 were used as described previously (Aubele *et al*, 2007). For further immunohistochemical analysis the following antibodies were applied (Table 1): p44/42 MAP kinase (Erk1 and Erk2), P-MAPK (detecting p44/42 MAPK phosphorylated on Thr202/Tyr204), PTEN (phosphatase and tensin homologue deleted on chromosome 10), and Sam68. Deparaffinisation of the TMA sections, antigen retrieval, and incubation with the primary antibody were performed as described (Quintanilla-Martinez *et al*, 2003). Staining and counterstaining were performed by an automated immunostainer (Ventana Medical System, Tucson, AZ, USA) (Quintanilla-Martinez *et al*, 2003; Aubele *et al*, 2007).

Tissue staining intensities were scored by two independent observers as described (Witton *et al*, 2003; Aubele *et al*, 2007) using a 4-point scale, where 0=no staining, 1=light staining, 2=moderate staining, and 3=strong staining. Immunohistochemical positivity for Sam68 was scored in percent positive stained tumor cells.

### Cell culture

T47D cells were obtained from the American Type Culture Collection and maintained in RPMI (Roswell Park Memorial Institute medium) supplemented with 10% foetal bovine serum (FBS), bovine insulin (10 *μ*g ml^−1^), and the antibiotics penicillin and streptomycin (0.25%) at 37°C in 5% CO_2_.

### Protein isolation, immunoprecipitation (IP), and western blot

For physical disruption of the cells, the T47D cell suspension was sonicated using high-frequency sound waves (3 × 30 s, power 30%). For protein isolation, the T-PER® Tissue Protein Extraction Reagent (Pierce, Rockford, IL, USA) was supplemented with Complete Mini-Protease inhibitor cocktail tablets (Roche, Mannheim, Germany), Phosphatase Inhibitor cocktail 1+2 (Sigma-Aldrich, Hamburg, Germany), and the extraction was performed according to the manufacturer's protocol.

For IP, the Seize® Primary Immunoprecipitation Kit (Pierce, Rockford, IL, USA) was applied according to the manufacturer's protocol with slight modifications. The primary antibody, PTK6, was coupled to AminoLink® Plus Coupling Gel at 22°C for 4 h with gentle end-over-end mixing. The antigen (protein lysate, 500 *μ*g) was precipitated at 22°C for 1.5 h with gentle end-over-end mixing. The precipitates were eluted using the ImmunoPure® elution buffer, containing 1% SDS to achieve dissociation of the antigen from the covalently immobilised antibody. The eluates were analysed by western blotting, using a standard concentration of 1 : 1000 of the primary antibody. To confirm the interaction of PTK6 and MAPK, an inverse IP with the immobilised MAPK-targeted antibody was carried out, and precipitated PTK6 was detected by western blot. To show that the interactions are due to a specific binding with PTK6, and not to an adsorption to beads, IP without antibody was also performed.

For western blot analysis, 20 *μ*g samples of protein lysate were resolved by SDS – polyacrylamide gel electrophoresis, and transferred to nitrocellulose membranes (Schleicher and Schuell, Dassel, Germany). Proteins were then detected with antibodies against PTK6, Sam68, HER2/neu, HER3, HER4, PTEN, MAPK, and phosphorylated MAPK (Table 1). The appropriate peroxidase-conjugated secondary antibodies (antirabbit NA934V, antimouse NA931V, 1 : 2000) were obtained from the GE Healthcare (Munich, Germany).

### Statistics

Correlations among the immunohistochemically evaluated markers, and between markers and clinical parameters, were examined by Spearman's rank correlation test. HER1 positivity was infrequent (only 6% of the cases), and was excluded from statistical analysis. Survival analysis was performed using disease-free interval for follow-up periods of 60, 120, and 240 months. The disease-free interval was defined as the interval from the date of surgery to the first locoregional recurrence and/or distant metastases.

For univariate survival analysis, Kaplan–Meier curves were calculated, and differences between strata were tested with the log-rank *χ*^2^-value. The multivariate analysis was performed using Cox proportional hazards regression and a combined stepwise selection algorithm (SAS Institute, Cary, NC, USA). All parameters reaching a significance level of *P*⩽0.15 in univariate analysis were offered to multivariate analysis. In all other tests, statistical significance was considered proven if *P*⩽0.05.

## Results

### PTK6 expression, its cooverexpression, and its correlation with other parameters

Two-thirds (69%, *n*=293) of the breast carcinomas showed a high PTK6 expression (IHC 2+, 3+; Table 2). In decreasing order, we found the overexpression of Sam68 (52%), MAPK (34%), PTEN (28%), and P-MAPK (in 7% of tumours). Most of these overexpressing tumours show co-overexpression with PTK6, as shown in (Table 2). The expression of PTK6 was not significantly correlated with clinical and histopathological parameters, such as lymph node status, tumour size, and histological grade or type (Table 3). Further, no association was found between PTK6 and HER2, HER3, and HER4 receptor expression. A significant correlation was observed between the expression of PTK6 and the expression of PTEN (*P*=0.002), Sam68 (*P*=0.0008), MAPK (*P*=0.01), and phosphorylated MAPK (*P*=0.001; Table 4).

### Prognostic relevance of parameters

#### Univariate analysis of parameters for a disease-free survival of patients

All clinical, histopathological, and immunohistochemical parameters were analysed for their prognostic relevance using Kaplan–Meier analysis. A significant inverse correlation with the disease-free survival of patients was found for lymph node status (*P*⩽0.0001), tumour size (*P*⩽0.0001), and the histological grade of tumours (*P*=0.002). A direct correlation (*P*⩽0.02) with the disease-free survival of patients was identified for PgR positivity and expression of HER4, PTK6, and Sam68 (Table 5). No significant survival effect was observed in univariate analysis for HER2/neu (0.08), HER3 (0.3), PTEN (0.2), MAPK (0.98), and P-MAPK (0.7), or for the ER status (0.2; Table 5).

#### Multivariate analysis

Multivariate analysis was used to assess the influence of markers on the disease-free survival of patients, together with that of the clinicohistopathological parameters. The stepwise selected parameters for a disease-free survival of patients of >240 months were lymph node status (risk factor 2.1), PTK6 expression (0.6), and tumour size (1.6; Table 6).

The prognostic value of key markers appeared to be time dependent (Aubele *et al*, 2007). Performing multivariate analysis for an interval of 60 months, the tumour size (risk factor 1.9), PTK6 expression (0.6), HER2/neu (1.0), and lymph node status (1.7) were independently prognostic. At an interval of 120 months, HER2/neu protein expression was no longer significant. Here, the stepwise selected parameters were lymph node status (risk factor 2.1), PTK6 expression (0.6), and tumour size (1.5).

#### The cohorts studied

Comparing the primary and the expanded tumour cohort with histopathological parameters showed that both cohorts contain different portions of ER- and PrR-positive tumours. Whereas the primary cohort contains 39% ER- and 33% PrR-positive tumours, the expanded cohort contains 79% ER- and 32% PrR-positive tumours. No prominent differences between the cohorts were found in relation to the lymph node status, tumour size, and the histological grade.

Performing multivariate analyses only with the newly added cases, the stepwise selected parameters for a disease-free survival of patients of >240 months were lymph node status (risk factor 2.3), tumour size (1.6), and PTK6 expression (0.7). This result is entirely in accordance with the results from the primary and the expanded cohort.

### Proteins coprecipitating with PTK6

**Figure 1:**
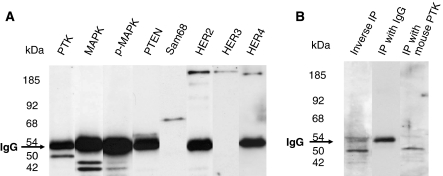
PTK6 coprecipitates with several signalling molecules in human breast cancer cell line, T47D (The specifity of single bands was proven using total cell lysates and western blot). (**A**) IP using the (rabbit) PTK6 antibody was analysed by western blot with rabbit antibodies specific for PTK, MAPK, P-MAPK, PTEN, HER2, and HER4, and mouse antibodies specific for Sam68 and HER3. (**B**) Inverse IP using rabbit antibody specific for MAPK was analysed by western blot with rabbit antibody specific for PTK6. IPs using rabbit IgG (sc-2027, Santa Cruz Biotechnology) and mouse PTK6 antibodies were analysed by western blot with the rabbit PTK6 antibody. No signals were detected by western blot after IP with the rabbit PTK6 antibody, which was blocked by PTK6 peptide (sc-1188P, Santa Cruz Biotechnology).

To analyse whether significant correlations among the expression level of different protein markers in tumour tissue are based on possible complex formation between these proteins, we performed IP with the PTK6 antibody using the T47D breast cancer cell line. Subsequent western blot analysis showed that PTK6 coprecipitated with MAPK, P-MAPK, HER2/neu, HER3, HER4, Sam68, and PTEN in T47D cell line (Figure 1). These results suggest that the correlations of IHC staining intensities in tumour tissues may represent molecular interactions between the corresponding proteins, as shown in T47D cell line.

## Discussion

In this study, we investigated protein expression of the cytoplasmic protein kinase PTK6 (BRK), the HER receptors, Sam68, PTEN, MAPK, and P-MAPK in breast carcinomas, and tested the associations between these markers and patient prognosis. We show that PTK6 protein expression is a prognostic marker for disease-free survival, on the basis of the data from a cohort of 426 invasive breast cancer cases. Further, we confirm the time dependence of the different prognostic parameters. The prognostic significance of HER receptors was only evident for short time period (60 months), as published previously, on the basis of the data from 105 breast carcinomas (Aubele *et al*, 2007). A similar time dependence of prognostic markers has also been reported by Tovey *et al* (2005) for the progesterone and HER1 – HER3 receptor expression in breast cancer.

The expression of PTK6 in breast carcinoma tissue was not associated with clinicohistopathological parameters, confirming our previous results (Aubele *et al*, 2007). In another study, however, PTK6 staining intensities were found to correlate with the histological grade of 250 breast carcinomas (Ostrander *et al*, 2007). Those discrepant findings may be caused by different compositions of the tumour cohorts.

A significant correlation between the protein expressions of PTK6 and HER2/neu, HER3, and HER4 was recently published (Aubele *et al*, 2007). The small cohort of tumour cases (*n*=105) used previously was now expanded to include 426 invasive breast carcinoma cases. The results presented here could not confirm a previously found significant correlation between PTK6 and HER receptor expression. This discrepancy in the results may also be due to differences in the composition of the cohorts. The cohort studied here contains proportionally more hormone-receptor-positive tumours. This may influence and change the statistical results, and is further supported by the novel finding that PTK6 expression correlates directly with the PrR status, which was not the case in our previous study.

The comparison of protein expression between the markers in tumour tissue showed that Sam68, PTEN, MAPK, and P-MAPK are all significantly correlated with PTK6 expression. To get an indication whether these statistical correlations of markers in tumour tissue may be caused by molecular associations with PTK6, we used IP and found that Sam68, PTEN, MAPK, and P-MAPK all coprecipitate with PTK6. Sam68 was originally identified as one of the major targets for PTK6 (Taylor and Shalloway, 1994; Derry *et al*, 2000; Babic *et al*, 2004; Haegebarth *et al*, 2004, 2005). It has been demonstrated that Sam68 is a substrate downstream of the EGFR (Lukong *et al*, 2005), and that tyrosine phosphorylation of Sam68 by PTK6 regulates negatively its RNA-binding activity (Derry *et al*, 2000; Babic *et al*, 2004; Haegebarth *et al*, 2004; Lukong *et al*, 2005). In our study, Sam68 expression was significantly associated with PTK6 and with PTEN expression, and inversely correlated with the expression of HER3 and HER4. Further, a direct correlation was identified between Sam68 expression and the disease-free survival of patients in univariate analysis.

PTEN was selected for our immunohistochemical study, because loss of PTEN expression showed correlation with disease-related death, lymph node metastasis (Depowski *et al*, 2001), and development of distant metastases in 88 tumour samples (Piekarski and Biernat, 2006). Positive PTEN expression in breast cancer tissue was associated with low tumour grade, longer survival time, and ER positivity (Winter *et al*, 2007). In our study, PTEN expression was correlated with the expression of PTK6 and with PrR and ER, confirming the results from Winter *et al* (2007). The association between PTK6 and PTEN expression in tissue corresponds to the coprecipitation of these proteins in the T47D cell line, indicative for a molecular association or complex formation of markers. The physiological meaning of this association must be analysed in more detail in future *in vitro* studies. No association could be identified between PTEN and the patients' prognosis, lymph node status, or tumour size.

Mitogen-activated protein kinase is known to be activated by HER receptors and to promote proliferation. Knockdown of PTK6-reduced proliferation (Harvey and Crompton, 2003) and Heregulin- and EGF-induced activation of Rac GTPase, ERK5, and MAPK (Ostrander *et al*, 2007). Gutierrez *et al* (2005)reported that high levels of active phosphorylated MAPK (p38MAPK) strongly correlated with upregulated HER2 and increased tamoxifen resistance in clinical specimens. In our study, we found a significant correlation between P-MAPK expression and the expression of PTK6, HER3, and HER4, but not with HER2. We further found a correlation between PTK6 expression and the levels of MAPK and P-MAPK in tumour tissues. A significant association, however, was found for PTK6, but not for MAPK or P-MAPK with patients' disease-free survival.

Although several interaction partners of PTK6 have been identified in cellular cultures, the signalling function of PTK6 as well as its function in breast cancer development and prognosis remains unclear. It is well accepted that the HER receptors transduce signals through associations with a variety of cytoplasmic target proteins containing SH2 and/or SH3 domains. PTK6 possesses both SH2 and SH3 domains (Kamalati *et al*, 1996; Zhang *et al*, 2005), and there is every reason to believe that it participates in HER receptor signalling processes. According to the current state of knowledge, however, association partners of PTK6 have been identified from all three main signal transduction pathways. It interacts and negatively regulates Akt (Kamalati *et al*, 2000; Zhang *et al*, 2005; Haegebarth *et al*, 2006). In addition, here, we could show that also PTEN, which belongs to the same PI3K/Akt signalling pathway, coprecipitates with PTK6 in T47D cell line, and is statistically associated with the expression of PTK6 in tumour tissue. PTK6 influences the MAPK pathway (as described above), and there is evidence that STATs are also physiological targets of PTK6 (Liu *et al*, 2006; Weaver and Silva, 2007). This obvious involvement of PTK6 in all three signalling pathways may be explained to some extent by cross talk between pathways. Moreover, PTK6 may function in post-transcriptional regulation of gene expression and through modulating the utilisation of messenger RNAs (Harvey and Crompton, 2004), thus influencing post-transcriptional activity of many other proteins. Therefore, elevated PTK6 expression in tumour tissue is presumably accompanied by alterations to the expression levels of multiple other molecules.

In conclusion, in this study, we show that PTK6 protein expression in 426 breast cancer cases is of high prognostic value, independent of the classical morphological and molecular markers, such as lymph node involvement, tumour size, and HER2 status. Although the role of PTK6 in breast cancer development and prognosis is not known, these results are of important clinical relevance as PTK6 may be a potential future target for the development of novel treatments for breast cancer. On the basis of this data and other expression studies, there is compelling reason to further investigate the cellular function and interaction pathways of PTK6.

## Figures and Tables

**Table 1:** Sources and dilutions of antibodies used for IHC and western blotting, respectively

		**Dilution**	
**Antibodies**	**Source**	**IHC**	**WB**
PTK6 (BRK; rabbit)	C-17(sc 1188), Santa Cruz Biotechnology, Heidelberg, Germany	1 : 100	1 : 5000
PTK6 (BRK; mouse)	5G1 (sc-66003), Santa Cruz Biotechnology, Heidelberg, Germany		1 : 200
HER1 (EGF-R; mouse)	31G7, Invitrogen, Heidelberg, Germany	1 : 50	
HER1 (EGF-R; rabbit)	H7298, DAKO, Glostrup, Denmark		1 : 50
HER2/neu (ErbB2; rabbit)	Hercep test™, K5204, DAKO, Hamburg, Germany	Ready for use	
HER2/neu (ErbB2; rabbit)	A0485, DAKO, Glostrup, Denmark		1 : 6000
HER3 (erbB3; mouse)	H3.105.5, Stratech, Suffolk, England	1 : 20	
HER3 (erbB3; mouse)	201P506A, Lab Vision Corp. Fremont, CA, USA		1 : 5000
HER4 (erbB4; mouse)	H4.77.16, Stratech, Suffolk, England	1 : 20	
HER4 (erbB4; rabbit)	4795, Cell Signaling Technology, Beverly, MA, USA		1 : 500
p44/42 MAP kinase (Erk1 and Erk2; rabbit)	4695, Cell Signaling Technology, Beverly, MA, USA	1 : 100	1 : 10000
p44/42 MAP kinase (rabbit)	9102, Cell Signaling Technology, Beverly, MA, USA		1 : 5000
Phosphorylated MAPK (rabbit)	9101, Cell Signaling Technology, Beverly, MA, USA	1 : 100	
Phosphorylated MAPK (rabbit)	4376, Cell Signaling Technology, Beverly, MA, USA		1 : 5000
PTEN (rabbit)	9559, Cell Signaling Technology, Beverly, MA, USA	1 : 50	1 : 2000
Sam68 (68 kDa protein; mouse)	sc-1238, Santa Cruz Biotechnology, Heidelberg, Germany	1 : 50	1 : 500

IHC=immunohistochemistry.

**Table 2:** Frequency of tumours (%) showing overexpression of markers, and co-overexpression between PTK6 and the other markers^a^

	**Highly expressed (%)**	**Co-overexpressed with PTK6 (% of highly expressed)**
PTK6	68.9	
PTEN	28.4	77.4
Sam68	52.1	73.9
MAPK	33.7	70.3
P-MAPK	6.5	94.4

IHC=immunohistochemistry; MAPK=mitogen-activated protein kinase; Sam68=Src-associated in mitosis 68 kDa.

a Thresholds for overexpression: PTK6, PTEN, MAKP, and P-MAPK; IHC ⩾2+; Sam68 IHC: ⩾50% positive tumour cells.

**Table 3:** Correlations between immunohistochemically assessed protein expressions and the clinicohistopathological parameters

	**Lymph node status**	**Histological grade**	**Histology**	**Tumour size**	**ER**	**PrR**
HER2/neu	0.16	**<0.0001**	**0.018**	0.19	**<0.0001**	**0.015**
		0.19	−0.11		−0.27	−0.13
HER3	0.13	0.15	0.3	**0.013**	**<0.0001**	**0.02**
				−0.06	−0.26	−0.12
HER4	0.3	0.01	0.1	0.8	**<0.0001**	0.5
		0.13			−0.22	
PTK6	0.8	0.5	0.9	0.5	0.2	**0.0004**
						0.18
Sam68	0.9	0.9	0.4	0.13	**<0.0001**	0.1
					0.21	
PTEN	0.5	0.11	0.4	0.4	**0.01**	**0.01**
					0.13	0.13
P-MAPK	0.8	0.1	0.7	**0.002**	0.1	**0.03**
				−0.18		0.12
MAPK	0.9	0.8	0.5	0.2	0.06	0.4
ER	0.5	**<0.0001**	0.8	0.2		**<0.0001**
		−0.20				0.27
PrR	0.1	**0.02**	0.1	0.2	**<0.0001**	
		−0.12			0.27	

ER=oestrogen receptor ; MAPK=mitogen-activated protein kinase; PrR=progesterone receptor; Sam68=Src-associated in mitosis 68 kDa.

For significant correlations, the *P*-values are printed in bold, and the rho factors are given.

Negative rho factors=inverse correlation.

**Table 4:** Correlations between immunohistochemically assessed markers

	**HER2/neu**	**HER3**	**HER4**	**PTK6**	**PTEN**	**Sam68**	**MAPK**	**P-MAPK**	**ER**
HER3	**<0.0001**								
	0.38								
HER4	**<0.0001**	**<0.0001**							
	0.29	0.65							
PTK6	0.17	0.8	0.3						
PTEN	0.5	0.1	0.4	**0.0016**					
				0.17					
Sam68	0.07	**0.0001**	**0.0001**	**0.0008**	**<0.0001**				
		−0.24	−0.21	0.18	0.22				
MAPK	0.15	**0.0018**	**<0.0001**	**0.01**	**0.011**	**0.033**			
		0.21	0.30	0.15	0.16	0.13			
P-MAPK	0.23	0.018	**<0.0001**	**0.001**	0.2	0.1			
		0.17	0.26	0.16					
ER	**<0.0001**	**<0.0001**	**<0.0001**	0.23	**0.01**	**<0.0001**	0.06	0.14	
	−0.26	−0.27	−0.22		0.13	0.21			
PrR	**0.015**	**0.02**	0.5	**0.0004**	**0.014**	0.11	0.37	**0.032**	**<0.0001**
	−0.12	−0.13		0.18	0.13			0.12	0.27

MAPK=mitogen-activated protein kinase; PrR=progesterone receptor; Sam68=Src-associated in mitosis 68 kDa.

For significant correlations, the *P*-values are printed in bold, and the rho factors are given.

Negative rho factors=inverse correlation.

**Table 5:** Results from univariate analysis of the single parameters for a disease-free survival of patients of ⩾240 months

**Parameter**	***P*-value**
Lymph node status (+/−)	***P*⩽0.0001**
Tumour size (⩽20, 20–40, >40 mm)	***P*⩽0.0001**
Histological grade	***P*=0.002**
Progesterone receptor	***P*=0.02** a
Oestrogen receptor	*P*=0.15
HER2/neu	*P*=0.08
HER3	*P*=0.25
HER4	***P*=0.028** a
PTK6	***P*=0.001** a
Sam68	***P*=0.018** a
MAPK	*P*=0.98
P-MAPK	*P*=0.7
PTEN	*P*=0.18

MAPK=mitogen-activated protein kinase.

Significant *P*-values are printed in bold.

a High expression corresponds to better prognosis.

**Table 6:** Results of the Cox multivariate regression analysis

**Parameter**	***P*-value**	**Coefficient**	**Relative risk**
*Total interval (*>*240 months):*			
Lymph node status (+/−)	0.0003	0.85	2.34
PTK6 expression	0.0058	−0.42	0.66^a^
Tumour size (−10, 10 – 20, >20 mm)	0.01	0.41	1.52
			Total *P*⩽0.0001
			
*Interval, 120 months:*			
Lymph node status (+/−)	0.0004	0.84	2.32
PTK6 expression	0.011	−0.40	0.67^a^
Tumour size (−10, 10 – 20, >20 mm)	0.013	0.41	1.50
			Total *P*<0.0001
			
*Interval, 60 months:*			
Tumour size (−10, 10 – 20, >20 mm)	0.0013	0.61	1.84
Lymph node status (+/−)	0.011	0.75	2.11
HER2/neu expression	0.024	0.0035	1.004
PTK6 expression	0.045	−0.384	0.681^a^
			Total *P*<0.0001

The summary of stepwise selected parameters is given for a disease-free survival of >240 months, 120 months, and 60 months.

Positive coefficients increase and negative coefficients reduce the risk of an event.

a High expression corresponds to better prognosis.
